# Screening for HPV-Related Oropharyngeal Cancer in Gay and Bisexual Men: Acceptability and Predicting Possible Use of “Oral Selfies” by Smartphone as a Secondary Prevention Approach

**DOI:** 10.3390/venereology2040016

**Published:** 2023-12-07

**Authors:** Michael W. Ross, Sarah L. Bennis, Niles Zoschke, Brian R. Simon Rosser, Cyndee L. Stull, Alan G. Nyitray, Samir S. Khariwala, Mark Nichols, Charlene Flash, Michael Wilkerson

**Affiliations:** 1 Institute of Sexual and Gender Health, Department of Family Medicine and Community Health, Medical School, University of Minnesota, Minneapolis, MN 55454, USA; 2 Division of Epidemiology and Community Health, School of Public Health, University of Minnesota, Minneapolis, MN 55454, USA;; 3 School of Public Health, University of Texas Health Science Center at Houston, Houston, TX 77030, USA;; 4 School of Dentistry, University of Minnesota, Minneapolis, MN 55455, USA;; 5 Department of Psychiatry and Behavioral Medicine, Medical College of Wisconsin, Milwaukee, WI 53202, USA;; 6 Department of Otolaryngology, Head and Neck Surgery, Medical School, University of Minnesota, Minneapolis, MN 55455, USA;; 7 Avenue360 Health Services, Houston, TX 77008, USA;

**Keywords:** HPV, Oropharyngeal cancer, gay/bisexual men, oral selfies, health belief model

## Abstract

Oropharyngeal cancers (OPCa) caused by HPV have emerged as one of the leading causes of malignancies caused by HPV infection. They are also significantly more likely to occur in males and in people with a history of oral sex with multiple partners. Gay and bisexual men are disproportionately affected by HPV-positive oropharyngeal cancers. We studied 1699 gay and bisexual men on 2 major dating sites in the US to assess their knowledge about HPV-related OPCa, attitudes toward screening for it, beliefs about oropharyngeal cancer screening based on the Health Belief Model, and attitudes toward possible screening approaches for OPCa. Knowledge on a 12-item scale was low, with a median of 5 items correct: 72% knew of the benefits of HPV vaccination. Significant predictors of needing OPCa screening included perception of risk for OPCa, seeing it as severe, having lower barriers, fewer reasons to avoid screening, higher knowledge, and being HPV vaccinated were significant predictors, explaining half the total variance. Most participants would accept routine, virtual/online doctor or dental appointments, and over half would accept an in-person screening. Nearly two-thirds stated that they would accept getting checked for OPCa if they could do self-screening at home, and half were prepared to use an online screening tool or app, where they could take an “oral selfie” and send it to a healthcare provider for examination. One-third stated that they would trust the results of a home screening completed by themselves and posted to a website equally as cancer screening completed online by a healthcare provider. Data indicate that despite low OPCA knowledge levels, the risk of HPV-associated OPCa was known. Being at personal risk and having knowledge of disease severity had 70% of the sample thinking about, or preparing to get, screening. Self-screening by a smartphone “oral selfie” transmitted to a screening website was acceptable to many gay and bisexual men, and online screening by a doctor or dentist was acceptable to most. OPCa screening in this population using electronic technology, together with the increasing incidence of HPV-associated OPCa in gay and bisexual men, brings together an opportunity to detect OPCa early.

## 1. Introduction

Oropharyngeal cancer (OPCa), and specifically oropharyngeal squamous cell carcinoma (OPSCC) risk, has historically been associated with high tobacco and alcohol use. However, carcinogenic human papillomavirus (HPV) infection has recently driven an increase in OPCa that now accounts for 71% of all OPSCCs in the US and 51% in the UK. Of these, 85–96% are caused by HPV-16 infections [1]. A recent study of 205 HIV-positive men who have sex with men (MSM) in the Czech Republic reported HPV in 96% of anal and 23% of oral samples, with HPV-16 being the most common genotype in both [2].

HPV (Human Papillomavirus) causes >70% of cases of oropharyngeal cancer (HPV-16, 18). Oropharyngeal (OP) cancer caused by HPV has a much better prognosis than classic OP cancer if detected and treated early. In a US study of the National Cancer Database, 2-year overall survival rates for HPV-positive cases vs. negative were 93.1% vs. 77.8% with an adjusted hazard ratio of 0.44 (95% CI, 0.36–0.53; *p* < 0.001) [3,4]. The highest risk of OP HPV-related cancer in men is oral sex with another man (OR = 8.89, 95% CI 2.14–36.8) [5]. Oropharyngeal HPV-related cancer is significantly higher in MSM compared with heterosexual men (3.78 times higher; 9.5%, 95% CI 3.7–15.2 vs. 2.9%, 95% CI 2.2–3.6) [6]. Lorenzoni et al. [7] report similar conclusions. Senkomago et al. [8], using US SEER data from 2012 to 2016, give rates of HPV-associated cancers of the cervix as 7.2 per 100,000, and HPV-associated cancers of the oropharynx in men as 8.5 per 100,000. While these may be referred to as “rare” cancers (defined by NCI as <15 per 100,000), given the history of HPV in causing cervical cancer, implying that any sexually transmissible cancer is “rare” has disturbing implications for reductions in the urgency of developing or modifying policy, vaccination, and screening approaches for sexually transmissible malignancies. Lechner et al. [1] indicate that the impact of HPV vaccination in men is unlikely to affect OPCa rates until 2045.

In the US and UK, the incidence of OPCa in men has surpassed that of cervical cancer in women. HPV-positive and HPV-negative OPSCCs are defined as separate entities, with distinct biological profiles, tumor characteristics, and outcomes. There are, however, considerable geographic and probably culturally associated prevalence rate differences [7], healthcare availability [9], and lifetime oral sex partner numbers which are strongly associated with HPV-positive OPSCC. While HPV emerges from the keratinized epithelium and there is epithelial contact during oral sex, semen is also known to carry HPV [10], as are spermatozoa specifically [11].

With tobacco, alcohol, and HPV as causes of OPCa, there is a confluence of risk factors in sexual and gender minority men (SGM) populations. SGM males have significantly higher rates of Alcohol-Related Disorder and Tobacco-Related Disorder than heterosexual males in nationally representative samples [12,13]. Thus, SGM are also at higher risk than heterosexual men of oral cancer and HPV-negative OPCa [6]. Given that males have typically had higher rates of OPCA than females, and SGM males have higher rates of alcohol and tobacco use than matched heterosexual men [14–16], this confluence of risk factors puts SGM men at the center of a dual epidemic of both OPCa and oral cancer cases.

Visual self-examination for cancer has decades of success and acceptance as a detection tool. In Australia, melanoma and diagnosis of other skin lesions by teledermoscopy has led to a national network of satellite-linked centers, with 3D Total Body Photography developed to detect skin lesions [17]. Self-screening and teledermoscopy are the norm, although barriers include forgetfulness to screen (44%), belief in low risk (25%), and low confidence in the ability to screen (25%) [17]. Wolf [18] looked at the specificity and sensitivity of four smartphone-based apps for melanoma and found that there was sensitivity of 98.1% and specificity of 93.7% for an app that sent data directly to a board-certified specialist. More recently, Wang et al. [19] reported that store-and-forward teledermatology (STF) melanoma images had comparable diagnostic and management accuracy to face-to-face consultation. However, smartphone apps that used computer algorithms had sensitivities as low as 6.8% and specificities as low as 30.4%. These data for melanoma images suggest that smartphone-acquired images have sufficient quality to permit good diagnoses, but that computer-based automated algorithms have inadequate predictive value. Fortunately, rapid information technology advances using convolutional neural networks (CNN) have brought algorithms to parity with clinical screening. In 2021, Perez and Ventura [20] found that CNN models attained better predictive performance than the state-of-practice models in melanoma diagnosis. It is apparent that visual self-examination and telescreening for some potential malignancies, combined with machine learning, are socially acceptable, and clinically have ethically and diagnostically acceptable sensitivity and specificity. Screening for oral malignancies is following this model.

Oral photographs have been investigated in low-income settings as a means of referral and review to oral medicine specialists. In India, Haron et al. [21] had dentists take oral photos with smartphones with cameras with varying resolutions. They reported that false-negative rates for the decision to refer the case to a specialist decreased as camera image resolution increased, concluding that the resolution of the image was best with 13-megapixel images (the highest used). Images were referred to dentists and oral medicine specialists who also gave the patients an oral examination. With the highest resolution camera, this teledentistry produced a false-negative rate of 0.13 and a false-positive rate of 0.00 for the decision to refer the case. Haron et al. [22] subsequently developed an app to facilitate mobile mouth screening, with a sensitivity of 94.0% and specificity of 95.5% for the decision to refer on to a specialist. Similarly, Perdoncini et al. [23] found that in comparing diagnoses from face-to-face and remote consultations, there was concordance in 92.7%. Acceleration of teleconsultation services for oral potentially malignant disorders has occurred as a result of the COVID-19 pandemic [24].

Photographs taken by patients can also be high quality: 91% of 23 participants in Cai et al.’s [25] study said that digital endoscope photographs and videos were easy to take, while 65% thought that smartphones were easy. For all methods of image acquisition, the time was <90 s. A blinded expert reviewer rated images that were representative of their oral examination as 14% for digital endoscopes, 50% for smartphone photographs, and for 55% for smartphone videos. Clinicians rated the smartphone videos as very acceptable for examination purposes (32%), compared with photographs (6%). These data, taken together, indicate that most smartphones can take high-quality photographs for oral screening and that, with training, high-quality photographs can be taken by either the clinician or the patient.

Simultaneously, information technology approaches to the detection and classification of oral lesions have also advanced. Welikala et al. [26] used deep neural networks to build automated systems to distinguish oral lesions from potentially malignant disorders (OPMD). They achieved a precision of 85% in identifying lesions, a precision of 67% in the identification of images that required referral, and the type of referral decision had a precision of 46% for low-risk OPMD referral and 65% for cancer/high-risk OPMD referral. More recently, Tanriver et al. [27] used deep learning to test the ability to detect oral lesions and classify them into categories (benign, OPMD, carcinoma) with a second-stage classification network. They reported that lesions of different types and sizes could be segmented with good precision using training images. With the best-performing model, they reported an Average Precision (AP) of 0.64 and an AP50 (50% true positive) of 0.95. Considerable progress is being made in developing automation for the identification of oral lesions that require referral.

A combination of oral imaging using white light plus autofluorescence imaging, and both smartphone-integrated and cloud-based CNNs, provided field-based validation by health workers in a major study of over 5000 subjects in both low-resource settings and tertiary hospitals in India [28]. Health workers used smartphone images of the mouth to diagnose oral pre-malignant and malignant lesions. Telediagnosis provided a sensitivity of 95% and specificity of 84%, and cloud-based CNN deep-learning architecture provided a sensitivity of 87% and specificity of 86%. While these were not self-obtained images, the study demonstrated that images obtained in the field combined with CNN deep learning can provide sensitivity and specificity equivalent to a diagnostic gold standard for oral malignancies.

Oral lesions differ in important ways from oropharyngeal lesions, which are more likely to be subcutaneous, and only those on the tonsils, uvula, and back of the throat are visible to a smartphone. On the other hand, they are in many cases visible and also potentially amenable to photographic screening.

Developments in electronic technology with smartphones and the concept of the “selfie”, along with recent developments in information and computer technology, have made it possible to engage disadvantaged communities. This is particularly important for SGM who are disproportionally impacted by oropharyngeal malignancies such as those caused by HPV-16. Innovative secondary prevention of cancers by screening and detection of early potentially malignant lesions in SGM is a new frontier in early clinical prevention of cancer in SGM populations.

While the protective effects of HPV vaccination will increase, it is estimated that the incidence of HPV-positive OPSCC will climb up to 2045, with meaningful reductions limited to those <56 years of age, where the effects of vaccination will begin to be seen [1]. Until then, investing in the development of novel early detection strategies is necessary to lower the substantial costs to human life [1]. Early self-detection by oral “selfies” is one such strategy.

Ross et al. [29] tested the hypothesis that gay and bisexual men would be able to take photos of sufficient quality to allow oropharyngeal screening [29]. From 1699 gay/bisexual men in the US surveyed about knowledge and attitudes to HPV-associated oropharyngeal cancer, a random sample of 320 men were invited to take an oropharyngeal “selfie” by smartphone and send it to the study website. Images were rated for quality by three healthcare professional raters blinded to each other’s ratings, with an otolaryngologist as the gold standard. In a second wave, those whose images were rated as unacceptable were sent a short instructional video and invited to send another image. Of the 65 invited, 46 did so. Following an instructional video made to train those who had poorer-quality images, additional quality images were received. One barrier, partial occlusion of the oropharynx by the tongue, remained. Quality oropharyngeal “selfies” are obtainable online. In total, 28.3% of these images were of clinically acceptable quality.

Given these preliminary data on feasibility, a second question to be asked is on acceptability, and levels of acceptability and interest in such an approach. We report on an investigation of Sexual Minority Men (SGM) to determine knowledge, attitudes, and beliefs regarding OPCa in SGM in the US, as well as the acceptability and feasibility of using “oral selfies” for OPCa screening.

## 2. Methods

This cross-sectional study aimed to recruit 1700 gay and bisexual men (SGM) from two online dating sites (Scruff and Jack’d; Perry Street Software Inc., New York, NY, USA) used by SGM. SGM with a profile on either site were shown a single advertisement with an embedded link to the survey during the recruitment period (February–March 2022). SGM aged 18 years or older who self-reported living in the US, having sex with a man in the past five years, and identified as a man were eligible to complete one online survey. Transgender men, non-binary persons, and other masculine-of-center individuals were also eligible to participate if they met the other criteria. Interested individuals were directed to a screener (Qualtrics, Provo, UT, USA) to determine eligibility. If deemed eligible, they continued to the consent process and consenting individuals were immediately able to access the survey.

The geo-targeted recruitment campaign was shown to all active users who logged in during the five-day campaign period in a certain area until the IRB-approved number of participants had responded to the invitation and had fully consented. Eligibility required being over the age of 18, being resident in the US or its territories, defining themselves as SGM, and having had sex with another man in the past 5 years. Participants could pause the survey and continue later, up to the time the maximum number of participants had been recruited. There were 4192 unique clicks onto Scruff and 5072 unique clicks onto Jack’d, 4464 people commenced the consent, 1836 completed the consent (19.86% of unique clicks), and 114 were removed during deduplication to give a final sample. After validation, deduplication, and internal consistency protocols had been completed, of the 1722 who remained, consented, and were eligible, 1699 completed the first question, comprising the study sample.

The recruitment algorithm was pre-programmed based on the ad run time and phased to hit specific geographic areas of the US throughout the ad run. All users who logged in while the run was active in their area would see the ad and it would be saved as an inbox message if they wished to check it out later. The link could only be accessed by Scruff/Jack’d users. The Scruff reach was 185,257 and made 417,296 total impressions. The Jack’d reach was 120,409 and made 247,956 total impressions.

Individuals who completed the survey were compensated USD 50. After the recruitment period, all surveys were reviewed to determine uniqueness using a modified validation and deduplication protocol [30]. Study materials were reviewed and approved by the University of Minnesota Institutional Review Board.

### 2.1. Demographics

All participants were asked their age, gender identity, sexual orientation, relationship status, race, ethnicity, education, income, health insurance status, and current zip code. Questions were based on language from the National Institutes of Health and the US Census. Additionally, participants reported select health information such as HPV vaccination status, COVID-19 vaccination status, and personal history of sexually transmitted infections (STIs).

### 2.2. Access to Healthcare

First, participants were asked how frequently they had heard of oropharyngeal cancer. At the end of the survey, participants were asked if they intended to ask a healthcare or dental professional to screen them for oropharyngeal cancer. We also included healthcare access questions such as affordability of healthcare and dental care, connection to a regular physician or dentist, personal history of oral or oropharyngeal cancer screening, history of examinations by an Ear, Nose, and Throat (ENT) specialist, and history of oral or oropharyngeal cancer diagnosis.

### 2.3. Knowledge, Attitudes, and Beliefs

To assess current knowledge of HPV infections, HPV vaccination, and oropharyngeal cancer, participants were asked 12 true–false knowledge questions. Twenty questions assessed attitudes towards healthcare and dental care, oropharyngeal cancer screening, and HPV vaccination. We used a five-point Likert scale (strongly agree, agree, neither agree nor disagree, disagree, and strongly disagree) for all attitude questions. Lastly, participants were asked 11 questions to assess beliefs regarding perceived oropharyngeal cancer risk, perceived oropharyngeal cancer severity, and the benefits and risks of oropharyngeal cancer screening, based on the Health Belief Model (HBM) [31], and intention to be screened for OPCa.

### 2.4. Hypothetical Screening

We designed a needs assessment of 16 items to inventory participants’ access to technology, healthcare and dental preferences, and level of comfort with taking a photo of their oral cavity, specifically the oropharynx (“oral selfie”). Acceptability questions included perceived quality of “selfies”, trust in self-screening tools, and thoughts towards sending “selfies” to different systems (i.e., their healthcare system, a screening application, a message board, or none of the above).

## 3. Analysis

Data were analyzed using SPSS version 27 and STATA IC16.1. Frequencies and percentages were calculated for the demographic data in Table 1.

Factor analysis was carried out on 10 items of the Health Belief Model (HBM) questions (Table 2). Principal components analysis followed by Direct Oblimin oblique rotation (Delta = 0) indicated, based on the Scree test and examination of the Factor Pattern Matrix, that there were three factors which met Kaiser’s criterion (more than three items per factor with loadings of >0.30). Factor scores were saved and used in an ordinal regression along with age, HPV vaccination status (vaccinated with at least one dose versus no vaccination or unsure if vaccinated), and the summed knowledge score (sum of true items on the twelve knowledge items, Table 3). Where distributions were skewed, non-parametric analyses (median and interquartile range) were used.

## 4. Results

A sample of 1699 SGM was recruited, provided consent, and completed at least one question. The median age was 39 (Interquartile Range 32–51) and racial and ethnic distributions approximated national distribution. Most (91.3%) were college educated, most (79.8%) identified as gay, and 17.8% as bisexual. A third had received HPV vaccination, and 17% reported being HIV positive (Table 1). More than half (56.6%) reported that they had smoked >100 cigarettes in their lifetime and 43.8% that they had smoked in the past 30 days. A large majority (84.0%) indicated that they had drunk alcohol at least once in the past year, and of these, 36% reported >1 drink per week.

Factor structure of the items characterizing constructs of the HBM indicated three factors: Risk perception of OPCa, Barriers to OPCa screening, and Benefits of screening and Severity of OPCa. These latent dimensions are consistent with the HBM, although Benefits and Severity were intercorrelated and emerged combined as a single factor (Table 2).

On one of the Barriers questions, 10.2% indicated Strongly agree or Agree with the question, “People are just trying to attack gay and bisexual men by frightening them about sexual health risks like oropharyngeal cancer” (the survey occurred two months before the 2022 Monkeypox epidemic was publicized in the media and so was not able to be influenced by that). Using the stages of change approach to determine where SGM are in their intentions to screen for OPCa, 5.9% said that they were “Already checking”; 22.4% “Preparing to check”; 56.3% “Thinking about checking”; and 15.4% “Not thinking about checking”.

Table 3 illustrates the 12 items of the knowledge scale. The mean score was 5.16 ± 3.19, Md = 5 correct answers out of a possible 12. However, 72% knew that HPV vaccination was available. Knowledge was not correlated *−*0.05 (*p* < 0.07) with age, indicating that age was not significantly associated with knowledge level, and there was no significant relationship of knowledge with race or ethnicity on ANOVA (F = 1.02, *df* = 6, *p* = 0.41).

Table 4 illustrates the predictors of feeling the need (or not) to be screened for OPCa. Most participants (57.5%) felt that they needed to be screened for OPCa, almost a third (30.2%) neither agreed nor disagreed, while 12.3% disagreed. The dimensions of the HBM that emerged as significant predictors of feeling a need (or not) to be screened were a perception of being at risk for OPCa; seeing it as a severe disease; and seeing lower barriers and lower reasons to avoid screening. An accurate knowledge of OPCa score on the 12-item scale (mean = 5.14, median = 5, SD = 3.19) was also a significant predictor, along with having had an HPV vaccination. Nearly one-third of the sample (30.8%) reported already being vaccinated with at least one dose, with an additional 16.4% responding “don’t know” or “not sure”.

Table 5 indicates that there were a number of OPCa self-screening possibilities considered. Over 85% would use a routine doctor or dental appointment virtually or online, and over half (55%) said they would use in-person screening. Sixty-three percent said that they would feel comfortable getting checked for OPCa if they could do a self-screening at home, and 45% would be prepared to use an online screening tool or app where they could take an “oral selfie” and send it to an oral healthcare provider for examination. One-third (33%) said that they would trust the results of a home screening completed by themselves as much as a cancer screening completed by a healthcare provider like a doctor or a dentist.

Almost all (99%) had a smartphone with a camera, and 78% had someone like a partner, roommate, or friend who could take a photo of the inside of their mouth if asked.

Attitudes toward OPCA screening were largely positive. Responding “Strongly agree” or “Somewhat agree”, 64% said that self-screening for OPCa was something that they would use, 65% would feel comfortable having someone they know take a picture of the inside of their mouth, and 60% were confident that they could take a clear picture of the inside of their mouths.

A majority (69%) said that free OPCa screening was needed, 34% said that they were worried about the cost of screening for OPCa, and 63% thought that there needed to be a website with more information on OPCa in gay and bisexual men. Of the sample, 91.3% had health insurance, and 74.8% had dental insurance. One in five (21%) said that they were afraid of dental examinations.

## 5. Discussion

These data were derived from a sample of gay and bisexual men recruited from two major US online dating sites. As such, they represent men who are sexually active with other men, and who responded to an online flyer for a study on OPCa in gay and bisexual men. The sample indicated good participation across race, ethnicity, and age, although it was heavily biased toward those with a college education.

Even with the high educational levels in the sample, the data show relatively low knowledge about HPV-16, consistent with other US findings [32]. The highest knowledge was that there is a vaccine available for HPV (72%) and that there is improved survival if the virus is detected early (54%). Thus, knowledge about the availability of the vaccine and its efficacy appears moderate. However, 63% of participants agreed that there is a need for a website with more information on OPCa in SGM. It is disturbing to note that over 10% agreed that information about health scares in SGM was part of an attempt to attack sexual and gender minorities. It seems that the lessons of the HIV epidemic are well remembered and that any perception of “weaponizing” health information may be counterproductive to screening and early prevention of disease. Data were collected just before the Monkeypox epidemic, so that would not have impacted the result.

Intentions to screen for OPCa indicated that of the sample, more than one in five were “Preparing to check” and more than half were “Thinking about checking”. Only 1 in 20 were “Already checking”. Even allowing for those who were thinking about checking being selectively attracted to the survey, these data indicate a widespread interest.

Finding predictors of accepting preventive screening given the widespread failure of people to accept disease preventives or screening tests for the early detection of asymptomatic disease in the 1950s led to the development of the Health Belief Model [30]. Using the four key constructs from the HBM as applied to screening for OPCa, the constructs of perceived severity, perceived risk, perceived susceptibility, and perceived benefits, almost one-third of the variance of intention to get screened for OPCa could be accounted for by these HBM constructs. Educating SGM communities using these HBM constructs would provide an efficient and effective model to promote HPV vaccination, along with the use of oral selfies if they can be demonstrated as a viable prevention approach, as part of increasing knowledge of available screening and prevention approaches.

Two-thirds of the men in this sample indicated that they would use self-screening for OPCa, and endorsed two approaches to screening: 85% an online screening with a doctor or a dentist, and 45% an online screening app. One-third would trust an online screening app as much as a screening completed by a healthcare provider such as a physician or dentist. Development of online screening tools and apps appears warranted, including not only the hardware but also the development of screening algorithms. Data from other areas of cancer screening, including for melanoma and oral cancer, have also shown that smartphone ownership and technology (including camera image clarity), and the development of deep-learning algorithms, can approach or meet acceptable standards of sensitivity and specificity for diagnosis [27,28]. The high proportion who would accept a virtual visit (which might be accomplished using smartphone images) suggests that this approach is a worthwhile step while awaiting studies on the development of deep-learning algorithms.

Tobacco use among gay men in large population-based samples in the US has been shown to be elevated compared to the general population [14–16], and so SGM are also at higher risk of oral cancer from these well-established risk factors. While HPV is rarely associated with oral cancer (as opposed to oropharyngeal cancer) [15], SGM’s higher rates of smoking, and alcohol use, make it appropriate to also consider oral cancer screening among this population. Our data should be considered relevant to attitudes and beliefs regarding oral cancer screening as well as OPCa screening.

### Limitations:

These data are not based on a random sample, and may not be representative of men using SGM dating sites in the US. They are, however, based on screen flyers rotated to all US states and territories and should be representative of population distribution. Because of the subject of the flyer, they are likely to be biased toward men who have some knowledge of HPV and OPCa. The sample was heavily biased toward college-educated men. Because of sample dropout over the course of the questionnaire, not all who started the questionnaire completed it to the end and received the incentive.

## 6. Conclusions

HPV-related OPCa is increasing in incidence in SGM but has high survival rates if detected and treated early: these data indicate a level of acceptability of the concept, and willingness to use oropharyngeal selfies as an early screening method. SGM have some knowledge of HPV-related OPCa but felt that a website with more information on OPCa was required, that the cost of screening was a barrier, and that screening should be offered. The HBM constructs of perceived risk, perceived severity, barriers, and benefits of treatment predicted 32% of intention to get screened for OPCa. However, routine screening by physicians and dentists in SGM is inconsistent. Early lesions in the visible oropharynx may be detectable by sight before becoming symptomatic. While HPV vaccination may prevent HPV-related OPCa, its full effect may not be apparent until 2045. SGM have indicated that they are enthusiastic about the possibility of smartphone-facilitated screening for OPCa early detection and pioneering approaches. Half of the participants agreed that they needed to be screened. A total of 85% of SGM were prepared to be screened by a virtual or online physician or dentist appointment, and 65% by self-screening online, with an “oral selfie” taken by themselves or a friend. One-third would trust a self-screening image as much as they would trust an in-person screening by a physician or dentist.

Take-home messages: (1) Data indicate that OPCa screening, and oral cancer screening more generally by “oral selfie” image, is a possibility widely accepted by US SGM participants in this study. (2) Recent successful use of machine learning technologies with oral cancer prevention suggests that it may be possible to expand innovative secondary prevention to identifying early-stage HPV-associated oropharyngeal lesions in SGM, who have a high risk of disease. (3) We can demonstrate in a preliminary trial that the production of “oral selfies” of a quality to make early diagnoses of possibly malignant OPCa is possible.(4) Next steps include determining ways of taking better images, using technology such as small self-lit videoscopes, and video rather than still images. (5) Early detection of HPV-related OPCa significantly decreases morbidity and mortality, and early and low-cost screening with AI could be cost-effective.

## Figures and Tables

**Table 1.** Demographic characteristics of participants (N = 1699).

	% (N)
What is your current age in years? (N = 1699)	
	39 (32–51) *
With which gender identity do you most identify? (N = 1684)	
Other	0.8% (14)
Cisgender man	95.2% (1604)
Non-binary, gender non-conforming	3.0% (50)
Transgender man	1.0% (16)
What is your sexual orientation? (N = 1690)	
Other	0.7% (12)
Bisexual	17.8% (300)
Gay	79.8% (1349)
Queer	0.9% (16)
Pansexual	0.8% (13)
What is your race? (N = 1657)	
White	66.3% (1098)
American Indian or Alaska Native	1.0% (17)
Asian	3.3% (54)
Black or African American	18.2% (301)
Native Hawaiian or other Pacific Islander	0.4% (6)
Other	3.6% (60)
Two or More Races	7.3% (121)
Are you Spanish/Hispanic/Latino? (N = 1673)	
No	83.9% (1404)
Yes	16.1% (269)
What is the highest level of education you have completed? (N = 1686)	
Associate's Degree	7.5% (127)
Bachelor's Degree	31.0% (522)
Graduate or Professional Degree	29.1% (491)
High School Graduate or GED	8.1% (136)
Less than High School	0.7% (11)
Some College but No Degree	23.7% (399)
What is your approximate yearly take-home income? This includes only your income. (N = 1207)	
	$50,000 (29,000–80,000) *
Do you currently have health insurance? (N = 1332)	
No	8.2% (109)
Yes	91.3% (1216)
Don’t know/not sure	0.5% (7)
Do you currently have dental insurance? (N = 1331)	
No	21.6% (287)
Yes	74.8% (995)
Don’t know/not sure	3.7% (49)
Have you received at least one dose of the human papillomavirus (HPV) vaccine? (N = 1625)	
Yes	32.7% (531)
No	49.9% (811)
Don’t know/not sure	17.4% (283)
HIV Status (N = 1374)	
HIV Negative	83.1% (1142)
HIV Positive	16.9% (232)a

* Continuous variables presented with the median and Interquartile Range.

**Table 2.** Health Belief Model structure.

Item	Loading
Factor 1: Risk Perception (N = 1471)	
Compared to the average person, I believe my risk of getting oropharyngeal cancer is: *	0.80
I believe I have several risk factors for oropharyngeal cancer	0.74
It is worthwhile checking for oropharyngeal cancer	0.36
Factor 2: Barriers (N = 1397)	
People are just trying to attack gay and bisexual men by frightening them about sexual health risks, like oropharyngeal cancer †	0.72
There are so many health hazards out there it is too exhausting to consider them all	0.67
I’m scared of what a healthcare provider (like a doctor or dentist) might find if I get screened for oropharyngeal cancer	0.58
I worry about the cost of screening for oropharyngeal cancer	0.57
Factor 3: Benefits and severity (N = 1353)	
Compared to other early screenings, I believe that the benefits of checking for oropharyngeal cancer are: *	0.73
I believe it will be easy for me to get screened for oropharyngeal cancer	0.69
Compared to other cancers, I believe that the severity of oropharyngeal cancer is: *	0.52

(Three factors explained 52.71% of total variance);

† data collected prior to the 2022 Monkeypox outbreak;

* five-point Likert scale, strongly agree to strongly disagree.

**Table 3.** Knowledge of OPCa.

Item	% Correct (N = 1333)
Oropharyngeal cancer is the same as oral (mouth) cancer	20.9
Oropharyngeal cancer includes cancer of the back of the tongue and the tonsils	50.7
Dentists cannot check for oropharyngeal cancer	46.2
There are two main types of oropharyngeal cancer, one caused by HPV-16 and, the other caused by heavy drinking and smoking	34.9
Young people of all genders can be vaccinated against HPV-related cancers	72.6
If caught early, HPV-related oropharyngeal cancer has a very high survival rate	54.4
Bad oral health (aching teeth and bleeding gums) can lead to oropharyngeal cancer	43.1
Oropharyngeal cancer is more common in women than in men	18.5
HPV-16 is the same virus that causes cervical cancer and most anal cancers	33.3
Oropharyngeal cancer is typically diagnosed through visual inspection of the mouth and throat	55.7
The widely available HPV vaccine series will protect against most types of oropharyngeal cancer if taken in adolescence and early adulthood	51.6
Oropharyngeal cancer is usually only detected only when it causes symptoms like problems swallowing or swelling in the neck	29.0

**Table 4.** Predictors of intention to get screened for oropharyngeal cancer.

Variable	Estimate	*SE*	Wald	*df*	Sig	95% CIs
Dependent variable: “I do not feel the need to be screened for oral or oropharyngeal cancer”						
Strongly agree	−5.59	0.53	113.16	1	<0.001	−6.62–−4.56
Agree	−3.92	0.51	58.21	1	<0.001	−4.92–−2.91
Neither agree nor disagree	−1.87	0.51	13.61	1	<0.001	−2.86–−0.88
Somewhat disagree	−0.51	0.52	0.95	1	<0.001	−1.52–0.51
Strongly disagree (Reference)						
Independent variables						
F1: Risk perception	−0.86	0.06	208.55		<0.001	−0.98–−0.74
F2: Barriers to Screening	0.62	0.06	111.45		<0.001	0.50–0.73
F3: Benefits of screening and Severity	−0.38	0.06	41.72		<0.001	0.50–−0.26
Knowledge score	−0.03	0.02	2.08		0.15	−0.06–0.009
Age	−0.00	0.01	0.22		0.64	−0.01–0.007
HPV vaccination status	−0.24	0.12	3.87		0.04	−0.48–−0.001

Regression model: Nagelkerke pseudo-R^2^ = 0.32; Pearson goodness-of-fit test, χ^2^ = 5891.43, *df* = 4942, *p* = 0.001.

**Table 5.** OPCa self-screening options, attitudes, and barriers.

Item	% (N = 1351)
Which of the following would you use to be screened for OPCa? (multiple answers possible)	
A routine doctor or dental appointment in-person?	55.3
A specialty dental or doctor appointment in-person	30.7
A routine dental or doctor appointment virtually or online	85.9
A specialty doctor or dentist appointment virtually or online	43.0
A community clinic	43.0
An LGBTQ+ specialty health clinic or center	29.3
An emergency dental practice	12.2
An emergency room	41.8
An online screening tool or app where you could take an oral selfie and send it to an oral healthcare provider for examination	45.3
I would feel comfortable getting checked for OPCa if I could do a self-screening at home	63.6
Do you have a phone with a camera? (Yes)	99.3
Do you have someone (like a partner, roommate or friend) that could take a photo of the inside of your mouth if asked? (Yes)	78.7
Response “Strongly agree” or “Agree”	
I would find oropharyngeal cancer screening too invasive	5.1
I think that self-screening for OPCa is something I would use	64.9
I would trust the results of a cancer screening I completed at home by myself just as much as a cancer screening completed by a healthcare provider (like a doctor or dentist)	33.0
I would feel comfortable having someone I did not know teach me how to take a picture of the inside of my mouth in-person	58.3
I feel comfortable having someone I know teach me how to take a picture of the inside of my mouth in-person	63.5
I would feel comfortable having someone I know take a picture of the inside of my mouth	65.0
I am confident I can take a clear picture (“selfie”) of the inside of my mouth	60.9
I do not feel the need to be screened for oropharyngeal cancer	9.6
I do not have enough time for OPCa screenings	37.4
There needs to be a website with more information on OPCa in gay and bisexual men	63.6
I worry about the cost of screening of OPCa	34.2
Free OPCa screening is needed	69.1
I am afraid of dental examinations	21.4

## Data Availability

Data are not available until completion of the study.
